# Erosive Effect of Acidic Beverages and Dietary Preservatives on Extracted Human Teeth—An *In Vitro* Analysis

**DOI:** 10.1055/s-0041-1742131

**Published:** 2022-04-18

**Authors:** Shivani Ramesh Maladkar, Priyanka Yadav, Archana Nayaka Akllemallenahalli Muniraja, Gayathri S. Uchil, Linet V. George, Dominic Augustine, Roopa S. Rao, Shankargouda Patil, Samudrala Venkatesiah Sowmya, Vanishri C. Haragannavar

**Affiliations:** 1 Department of Oral Pathology and Microbiology, Faculty of Dental Sciences, M.S. Ramaiah University of Applied Sciences, MSR Nagar, Bengaluru, Karnataka, India; 2 College of Dental Medicine, Roseman University of Health Sciences, South Jordan, Utah, USA; 3 Centre of Molecular Medicine and Diagnostics (COMManD), Saveetha Dental College and Hospitals, Saveetha Institute of Medical and Technical Sciences, Saveetha University, Chennai, India

**Keywords:** erosion, acidic beverages, demineralization, dental hard tissues, dietary preservatives, polarizing microscope, stereomicroscope

## Abstract

**Objectives** Frequent consumption of acidic beverages and dietary preservatives in younger generation, diet-conscious (celebrities), and obese individuals have a rapid impact on demineralization of the teeth. An attempt was made to analyze the erosive potential of various acidic beverages.

**Materials and Methods** One hundred and ninety extracted human permanent teeth were sectioned longitudinally, pre-weighed, randomly grouped, and placed in nine acidic beverages (200 mL) with predetermined pH, i.e., three commercially available fruit juices, three carbonated drinks, and three dietary preservatives.

**Statistical Analysis** The sectioned specimens ( *n* = 10) were analyzed at time intervals of 12, 24, 48, and 96 days. Mean weight loss was calculated, and surface changes were assessed under a stereomicroscope. The demineralization pattern and microscopic changes were observed under a compound and polarizing microscope. One-way analysis of variance test followed by Tukey's post-hoc analysis was employed.

**Results** Overall the maximum demineralizing effect was caused by vinegar and apple cider. In the fruit juices category, lemon juice induced significant changes, while in the carbonated drinks category Coca-Cola induced the maximum changes and in the category of food preservatives vinegar induced the maximum changes. Severe discoloration was seen with respect to Coca-Cola followed by Mountain Dew (carbonated drink).

**Conclusion** The present study is unique as three different types of microscopes have been employed and both dentin and enamel of permanent teeth have been analyzed. In addition, the effect of dietary preservatives on hard tissues was evaluated. Oral health educators can reinforce important practices such as decreasing the frequency of consumption and time duration of beverage contact with the teeth. Also, the use of mouth rinses and buffering agents after the consumption of dietary beverages can be advocated along with regular fluoride application for those who are regular consumers.

## Introduction

Erosion is the chemical dissolution of the tooth structure due to acids. A process that reduces the mineral content of any tissue is called demineralization. The hardness and strength of the tooth enamel is greatly influenced by demineralization and remineralization processes. Due to the loss of the mineral content, dental erosion occurs. 1 It is a superficial phenomenon which eventually causes the dissolution of subsurface layers. Due to the loss of mineralized content, the tooth becomes more susceptible to the attack of bacteria leading to the initiation of caries, if not treated on time can lead to the loss of the complete tooth structure. 2 Consumption of acidic beverages causes an acidic environment in the oral cavity which leads to the thinning of enamel due to demineralization. The buffering activity of saliva is also not effective as these acidic beverages have varying amounts of resistance against it. 3 4 Not only extrinsic drinks but intrinsic acidic sources can also cause erosion. Bulimia nervosa, anorexia nervosa, gastroesophageal reflux disease, etc., are a few examples of intrinsic acid-producing conditions which lead to the demineralization of the tooth structure. Acids in the mouth lower the pH and, thus, lead to the loss of minerals from the tooth surface finally leading to the exposure of dentin resulting in sensitivity, pain, and loss of tooth structure. 5 6

Fruit juices, aerated drinks, and carbonated drinks are a major part of todays diet globally. Also, the change in lifestyle habits of the general population, addiction to video games, and long television hours have influenced a lot of people to consume these drinks frequently for an extended time. Sedentary lifestyle habits allow the carbonated drinks to remain in contact with the dental hard tissues for a longer period leading to decreased salivary activity and loss of enamel at a very fast rate. 7 8

Hence, the present study aims to evaluate the effect of acidic dietary beverages on the macroscopic and microscopic structures of enamel and dentin. The objectives of the study include (1) estimating the mean weight reduction in the tooth structure on exposure to acidic beverages, (2) analyzing the surface characteristic changes in enamel and dentin following exposure to acidic beverages, (3) examining the microscopic changes in ground sections of enamel and dentin on exposure to acidic beverages.

Not many studies have used both macroscopic and microscopic techniques simultaneously to study and demonstrate the effect of both acidic beverages and dietary preservatives on the tooth (enamel and dentin). Information on the effect of dietary preservatives has also been provided.

## Materials and Methods

The study was conducted at the Department of Oral Pathology and Microbiology, Ramaiah University of Applied Sciences, Bangalore, Karnataka, India. Verbal and written ethical consent was obtained from the patients stating that following extraction their tooth/teeth would be used for research purposes, and it was also reassured to patients that their identity would not be revealed. The present study analyzed the microscopic and macroscopic changes in the structure of enamel and dentin on exposure to acidic beverages with the use of anterior and posterior extracted human teeth. The criteria for inclusion were teeth requiring extraction for orthodontic reasons only which are intact without restorations, fracture, or dental caries. Teeth extracted due to fracture, decay, and failed restorations were excluded.

- *Sample Preparation* : Twenty teeth specimens were allocated for each test group (nine test groups) and 10 teeth for one control group, a total of 190 teeth (180 test and 10 control) were considered for analysis. The teeth were vertically split, using a diamond bur to expose the enamel and dentin, into sections (each of these sections was weighed before and after exposure). At each of the four-time intervals, 10 sections were taken out for analysis, five were viewed under stereomicroscope and five were subjected to ground section. Prior to the start of the experiment, the sliced specimens were weighed using an electric precision balance and the pH of the acidic beverages was measured using a digital pH meter (pHep, range 0.0–14.0, resolution 0.1 pH, accuracy at 20°C/68°F is ± 0.1 pH). The pH levels of the nine test samples recorded were as follows: orange juice (5.1), pineapple juice (4.4), lemon juice (4.2), Coca-Cola (3.5), Mountain Dew (4.1), Red Bull (4.4), vinegar (3.5), apple cider (3.7), and tomato ketchup (4.6).
- *Exposure to Test Samples* : The split specimens were placed in 200 mL of the following acidic beverages: commercially available orange juice, lemon juice, pineapple juice, Coca-Cola, Mountain Dew, Red Bull, apple cider, vinegar, and tomato ketchup. The tooth sections were removed from these acidic beverages at an interval of 12, 24, 48, and 96 days for macroscopic and microscopic analyses. The solutions were changed at each time interval. The teeth in the control group were immersed in distilled water of pH 7.
- *Post-Exposure Weight Analysis* : The specimens were weighed using a digital weighing balance, and the mean weight loss before and after exposure was calculated.
- *Microscopy* : The sectioned specimens were viewed under a stereomicroscope to assess and analyze the degree of surface changes (roughness and discoloration). Ground sections of the specimens (40–60 μm in thickness) were made using Arkansas stone, and these sections were viewed under both bright field microscope and polarizing microscope. Under the compound microscope, the parameters assessed were the changes in the integrity of dentinal tubules and the striae of Retzius. The ground sections were prepared at each time interval for analysis.
Parameters assessed under the polarizing microscope were the variation and concentration of color with distribution of birefringence which corresponds to the demineralization each specimen has undergone after the specific intervals of exposure to the individual acidic beverages. A customized scoring criterion from 0 to 3 was developed based on the severity of changes for the parameters analyzed ( Table 1 ).
- **Interpretation and Statistical Analysis:** The scores for each experiment conducted were tabulated. The difference in pre- and post-exposure weights of specimens and different scores obtained under stereomicroscope, bright field microscope, and polarizing microscope were tabulated and analyzed statistically for significance. The scoring criteria designed are presented in the table. The test extracts were labeled as follows: 1 = orange juice, 2 = pineapple juice, 3 = lemon juice, 4 = Coca-Cola, 5 = Mountain Dew, 6 = Red Bull, 7 = vinegar, 8 = apple cider, and 9 = tomato ketchup.

**Table 1:** Customized tri-microscopic scoring criteria for interpretation of macroscopic and microscopic findings of demineralization changes caused by acidic beverages

Parameters		0	1	2	3
Discoloration		No	Mild	Moderate	Severe
Stereomicroscope	Surface irregularities	No roughness	Mild with grainy appearance	Moderate with craze lines	Severe with chalky/sandstone appearance
Compound microscope	Striae of Retzius	Distinct and regular	Faint and regular	Faint and irregular	Indistinct
	Dentinal tubules	Distinct and continuous	Faint and continuous	Faint and discontinuous	Sparse
Polarizing microscope	Demineralization	Organized	Organized with irregular borders	Disorganized	Disorganized and patchy

## Results

**Fig. 1:**
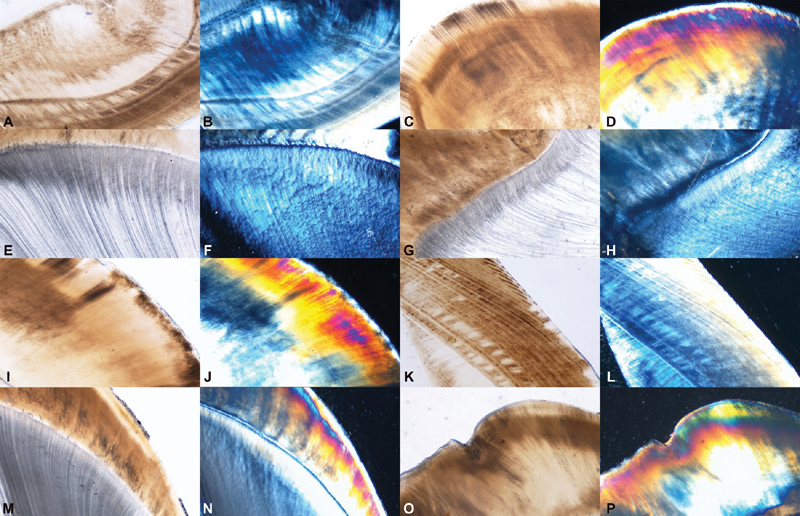
( **A** – **P** ): Photomicrographs of control sections of teeth. The integrity of striae of Retzius and dentinal tubules were well maintained. The polarizing microscope revealed no patchy or demineralized areas. The birefringent colours were well organized and uniform in distribution.

**Fig. 2:**
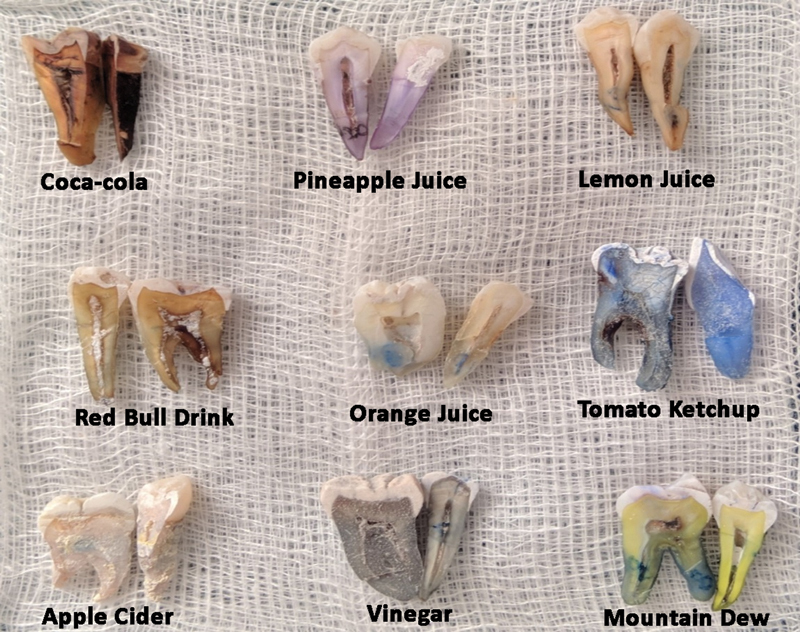
Macroscopic view of discolored teeth. Coca-Cola showed maximum discoloration followed by Mountain Dew and Red Bull drink. Tomato ketchup showed least discoloration followed by orange juice ( *bluish tinge due to labeling ink used* ).

**Fig. 3:**
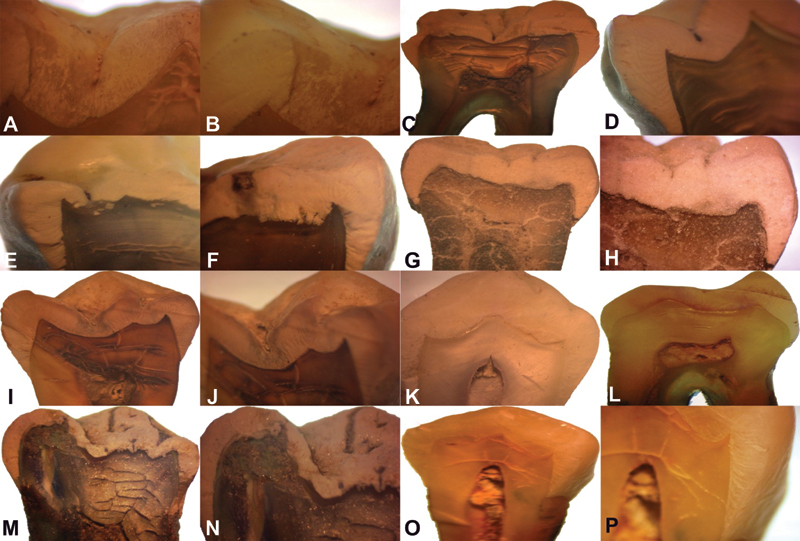
Stereomicroscope photomicrographs of sectioned teeth immersed in ( **A** – **D** ) apple cider and ( **E** – **H** ) vinegar showed maximum surface irregularities, loss of gloss, and roughness at 48 days. Changes observed in ( **I** and **J** ) Coca-Cola, ( **K** ) lemon juice, and ( **L** ) Mountain Dew at 48 days. ( **M** and **N** ) Maximum changes induced by vinegar at 96 days. ( **O** and **P** ) Tomato ketchup not exhibiting any noticeable changes.

**Fig. 4:**
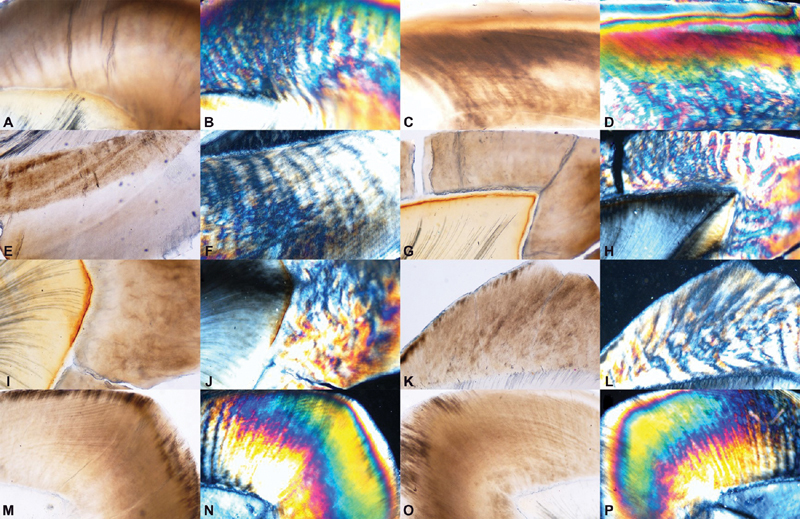
Bright field microscope photomicrographs showing loss of integrity of striae of Retzius at 96 days. Polarized microscopy photomicrographs of the same sections showing loss of stratification of birefringent colours, patchy and haphazard birefringence. Apple cider (A–B), vinegar (C–D), coca–cola (E–F), red bull drink (G–H), mountain dew (I–J), lemon juice (K–L). No significant changes observed in orange juice (M–N) and tomato ketchup which show good integrity of striae of Retzius with uniform and stratified distribution of birefringent colours (O–P).

**Fig. 5:**
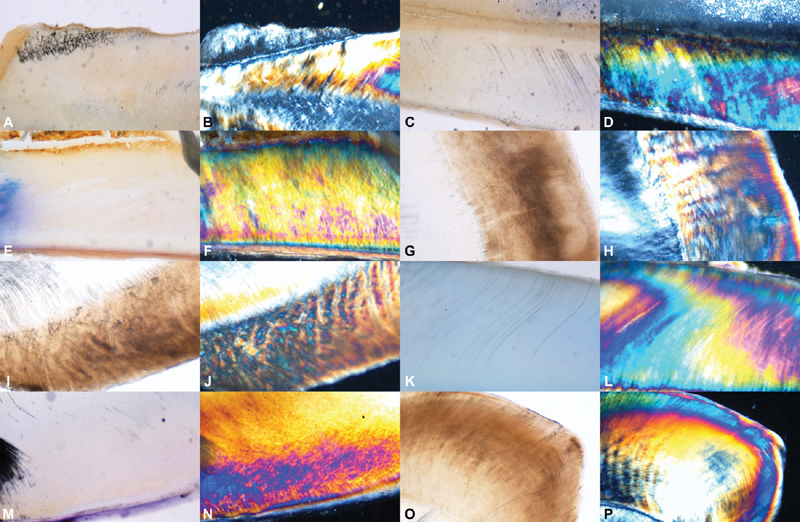
Bright field microscope photomicrographs showing loss of integrity of dentinal tubules at 96 days. Polarized microscopy photomicrographs of the same sections showing loss of stratification of birefringent colours, patchy and haphazard birefringence. Apple cider (A–D), vinegar (E–F), Coca–cola (G–H), red bull drink (I–J), mountain dew (K–L). No significant changes observed in tomato ketchup (m–n) and orange juice (O–P) which show good integrity of dentinal tubules and striae of Retzius with uniform and stratified distribution of birefringent colours.

- *Control Teeth*
The control sections did not show any mean weight change at any given time intervals. No discoloration or surface irregularities were observed. The integrity of striae of Retzius and dentinal tubules were well maintained. The polarizing microscope revealed no patchy or demineralized areas. The birefringent colors were well organized and uniform in distribution, ( Fig. 1A–P ).
- *Mean Weight Reduction*
For all nine extracts, the mean weight change at 48- and 96-day time intervals was significantly higher compared with that at 12- and 24-day time intervals ( *p* < 0.001), ( Table 2 ). Vinegar (sample 7) showed the maximum reduction in weight compared with all other extracts, and the weight reduction was significant between the 12- and 96-day exposure as well. Apple cider also showed moderately high values for weight change followed by lemon juice.
- *Discoloration After Exposure*
The comparison of mean discoloration values between different test samples at 96-day time interval indicated a significant difference in the mean discoloration values between the nine study samples and between the samples and control ( *p* < 0.001), ( Table 3 ). Multiple comparisons between the extracts demonstrated that Coca-Cola, Mountain Dew, and Red bull had maximum discoloration capacity when compared with pineapple juice, lemon juice, and vinegar which showed the least mean discoloration scores ( *p* < 0.001). This was followed by apple cider, tomato ketchup, and orange juice, ( Fig. 2 ).
- *Stereomicroscopic Analysis*
The comparison of mean surface irregularities between different test samples at 96-day time interval showed a significant difference in the mean scores between the nine study samples and between samples and control ( *p* < 0.001), ( Table 4 ). Vinegar and apple cider showed maximum surface irregularities, loss of gloss and roughness, followed by lemon juice, Coca-Cola, and Mountain Dew. Tomato ketchup did not show any appreciable changes compared with normal, ( Fig. 3A–P ).
- *Brightfield Microscopic Analysis*
The mean values for integrity of striae of Retzius scores significantly increased at 96-day time interval as compared with other time lines ( *p* < 0.001), ( Table 5 ), whereas the mean of integrity of dentinal tubules scores showed a significant increase at 24 and 48-day time intervals itself, as compared with 12-day time interval ( *p* < 0.001). Maximum loss of integrity of striae of Retzius was observed in teeth exposed to apple cider and vinegar, followed by Coca-Cola and Red Bull drink. Lemon juice showed significant changes at 96 days. Orange juice and tomato ketchup did not show any changes compared with normal, ( Fig. 4A–P ).
Maximum loss of integrity of dentinal tubules was observed in teeth exposed to vinegar and apple cider ( Table 6 ). This was followed by Coca-Cola, Red Bull, Mountain Dew, and lemon juice with similar values. Again, the least changes were induced by tomato ketchup, only at 96 days.
- *Polarizing Microscopic Analysis*
The mean values for degree and pattern of demineralization scores significantly increased at 96-day time interval as compared with other time lines for all test samples ( *p* < 0.001) ( Table 7 ). Demineralized areas were observed as patchy and haphazard areas of birefringence with loss of stratification of colors in contrast to normal tooth where the birefringent colors were stratified and arranged in an organized fashion. The degree and pattern of demineralization were found to be high once again for vinegar and apple cider closely followed by Red Bull and lemon juice. Coca-Cola and Mountain Dew had comparable values at 96 days. The least changes were induced by tomato ketchup and orange juice, ( Fig. 5A-P ).

**Table 2:** Comparison of mean weight change (in grams) between different extracts at 96-day time interval using one-way ANOVA test followed by Tukey's post-hoc analysis

Test samples	*N*	Mean	SD	Min	Max	*p* -Value	Sig. Diff.	*p* -Value
Orange juice	10	0.040	0.019	0.01	0.07	<0.001 ^*^	E1 vs. E2, 7 and 8	<0.001 ^*^
Pineapple juice	10	0.120	0.026	0.08	0.16		E2 vs. E3, 4, 5, 6, 7, 8, and 9	<0.001 ^*^
Lemon juice	10	0.030	0.009	0.02	0.05		E3 vs. E4, 6, 7, and 8	<0.01 ^*^
Coca-Cola	10	0.060	0.014	0.04	0.08		E4 vs. E7, 8 and 9	<0.001 ^*^
Mountain Dew	10	0.040	0.019	0.01	0.07		E5 vs. E7 and 8	<0.001 ^*^
Red Bull	10	0.060	0.017	0.03	0.09		E6 vs. E7, 8 and 9	<0.001 ^*^
Vinegar	10	0.430	0.026	0.39	0.47		E7 vs. E8 and 9	<0.001 ^*^
Apple cider	10	0.210	0.020	0.18	0.24		E8 vs. E9	<0.001 ^*^
Tomato ketchup	10	0.020	0.009	0.01	0.03		–	–

Abbreviations: ANOVA, analysis of variance; E, extract; Max, maximum; Min, minimum; SD, standard deviation, Sig. Diff., significant difference.

*denotes significant *p* -Values.

**Table 3:** Comparison of mean discoloration values between different extracts at 96-day time interval using one-way ANOVA test followed by Tukey's post-hoc analysis

Test samples	*N*	Mean	SD	Min	Max	*p* -Value	Sig. Diff	*p* -Value
Orange juice	10	1.90	0.32	1	2	<0.001 ^a^	E1 vs. E2, 3, 4, 5, and 7	<0.001 ^a^
Pineapple juice	10	0.00	0.00	0	0		E2 vs. E4, 5, 6, 8, and 9	<0.001 ^a^
Lemon juice	10	0.00	0.00	0	0		E3 vs. E4, 5, 6, 8, and 9	<0.001 ^a^
Coca-Cola	10	3.00	0.00	3	3		E4 vs. E6, 7, 8, and 9	<0.001 ^a^
Mountain Dew	10	3.00	0.00	3	3		E5 vs. E6, 7, 8, and 9	<0.001 ^a^
Red Bull	10	2.00	0.00	2	2		E6 vs. E7	<0.001 ^a^
Vinegar	10	0.00	0.00	0	0		E7 vs. E8	<0.001 ^a^
Apple cider	10	1.80	0.42	1	2		–	–
Tomato ketchup	10	1.80	0.42	1	2		–	–

Abbreviations: ANOVA, analysis of variance; E, extract; Max, maximum; Min, minimum; SD, standard deviation, Sig. Diff., significant difference.

**Table 4:** Comparison of mean Surface Irregularity values between different extracts at 96-day time interval using one-way ANOVA test followed by Tukey's post-hoc analysis

Test samples	*N*	Mean	SD	Min	Max	*p* -Value	Sig. Diff	*p* -Value
Orange juice	10	1.00	0.47	0	2	<0.001 ^a^	E1 vs. E2, 4, 5, 7, and 8	<0.001 ^a^
Pineapple juice	10	2.80	0.42	2	3		E2 vs. E3, 4, 5, 6, and 9	<0.001 ^a^
Lemon juice	10	1.00	0.00	1	1		E3 vs. E4, 5, 7, and 8	<0.001 ^a^
Coca-Cola	10	2.00	0.00	2	2		E4 vs. E6, 7, 8, and 9	<0.001 ^a^
Mountain Dew	10	2.00	0.00	2	2		E5 vs. E6, 7, 8, and 9	<0.001 ^a^
Red Bull	10	1.00	0.00	1	1		E6 vs. E7 and 8	<0.001 ^a^
Vinegar	10	3.00	0.00	3	3		E7 vs. E9	<0.001 ^a^
Apple cider	10	3.00	0.00	3	3		E8 vs. E9	<0.001 ^a^
Tomato ketchup	10	0.90	0.32	0	1		–	–

Abbreviations: ANOVA, analysis of variance; E, extract; Max, maximum; Min, minimum; SD, standard deviation, Sig. Diff., significant difference.

**Table 5:** Comparison of mean integrity striae of Retzius values between different extracts at 96-day time interval using one-way ANOVA test followed by Tukey's post-hoc analysis

Test samples	*N*	Mean	SD	Min	Max	*p* -Value	Sig. Diff	*p* -Value
Orange juice	10	0.90	0.32	0	1	<0.001 ^a^	E1 vs. E3, 4, 6, 7, 8, and 9	<0.001 ^a^
Pineapple juice	10	1.00	0.00	1	1		E2 vs. E3, 4, 6, 7, 8, and 9	<0.001 ^a^
Lemon juice	10	1.90	0.32	1	2		E3 vs. E5, 7, 8, and 9	<0.001 ^a^
Coca-Cola	10	1.90	0.32	1	2		E4 vs. E5, 7, 8, and 9	<0.001 ^a^
Mountain Dew	10	0.90	0.32	0	1		E5 vs. E6, 7, 8, and 9	<0.001 ^a^
Red Bull	10	1.90	0.32	1	2		E6 vs. E7, 8, and 9	<0.001 ^a^
Vinegar	10	3.00	0.00	3	3		E7 vs. E9	<0.001 ^a^
Apple cider	10	3.00	0.00	3	3		E8 vs. E9	<0.001 ^a^
Tomato ketchup	10	0.00	0.00	0	0		–	–

Abbreviations: ANOVA, analysis of variance; E, extract; Max, maximum; Min, minimum; SD, standard deviation, Sig. Diff., significant difference.

**Table 6:** Comparison of mean integrity of dentinal tubules between different extracts at 96-day time interval using one-way ANOVA test followed by Tukey's post-hoc analysis

Test samples	*N*	Mean	SD	Min	Max	*p* -Value	Sig. Diff	*p* -Value
Orange juice	10	1.00	0.00	1	1	<0.001 ^a^	E1 vs. E3, 4, 5, 6, 7, and 8	<0.001 ^a^
Pineapple juice	10	1.00	0.00	1	1		E2 vs. E3, 4, 5, 6, 7, and 8	<0.001 ^a^
Lemon juice	10	1.90	0.32	1	2		E3 vs. E7, 8 and 9	<0.001 ^a^
Coca-Cola	10	2.00	0.00	2	2		E4 vs. E7, 8 and 9	<0.001 ^a^
Mountain Dew	10	1.80	0.42	1	2		E5 vs. E7, 8 and 9	<0.001 ^a^
Red Bull	10	1.90	0.32	1	2		E6 vs. E7, 8, and 9	<0.001 ^a^
Vinegar	10	3.00	0.00	3	3		E7 vs. E9	<0.001 ^a^
Apple cider	10	3.00	0.00	3	3		E8 vs. E9	<0.001 ^a^
Tomato ketchup	10	0.80	0.42	0	1		–	–

Abbreviations: ANOVA, analysis of variance; E, extract; Max, maximum; Min, minimum; SD, standard deviation, Sig. Diff., significant difference.

**Table 7:** Comparison of mean demineralization values between different extracts at 96-day time interval using one-way ANOVA test followed by Tukey's post-hoc analysis

Test Samples	*N*	Mean	SD	Min	Max	*p* -Value	Sig. Diff	*p* -Value
Orange juice	10	1.00	0.00	1	1	<0.001 ^a^	E1 vs. E3, 4, 5, 6, 7, and 8	<0.001 ^a^
Pineapple juice	10	1.00	0.00	1	1		E2 vs. E3, 4, 5, 6, 7, and 8	<0.001 ^a^
Lemon juice	10	2.80	0.42	2	3		E3 vs. E4, 5 and 9	<0.001 ^a^
Coca-Cola	10	1.90	0.32	1	2		E4 vs. E6, 7, 8, and 9	<0.001 ^a^
Mountain Dew	10	1.80	0.42	1	2		E5 vs. E6, 7, 8, and 9	<0.001 ^a^
Red Bull	10	2.90	0.32	2	3		E6 vs. E9	<0.001 ^a^
Vinegar	10	3.00	0.00	3	3		E7 vs. E9	<0.001 ^a^
Apple cider	10	3.00	0.00	3	3		E8 vs. E9	<0.001 ^a^
Tomato ketchup	10	1.00	0.00	1	1		–	–

Abbreviations: ANOVA, analysis of variance; E, extract; Max, maximum; Min, minimum; SD, standard deviation, Sig. Diff., significant difference.

To summarize the results, vinegar showed the maximum weight change post-exposure. Coca-Cola and Mountain dew showed the highest mean discoloration scores compared with other test samples. Vinegar and apple cider showed maximum surface irregularities and roughness viewed under stereomicroscope. Vinegar and apple cider were found to induce maximum changes in striae of Retzius and dentinal tubules. Maximum degree and pattern of demineralization were also observed for vinegar and apple cider under polarizing microscope.

## Discussion

India's soft drink consumption has grown by 87% in the past 9 years, and the net increase in the next 3 years is expected to be approximately 7%. Statistics show that the per capita consumption of soft drinks was 4.1 L in 2018 and is expected to be 4.6 L by 2021. 9 Citric, phosphoric and carbonic acids are the three main dietary acids present in these soft drinks. Additionally, acetic acid is present in preservatives like vinegar and apple cider. Hence, the aim of this study was to analyze the relative cumulative effect of erosivity for the selected acidic beverages with an emphasis on dietary preservatives. These data will allow a ranking for the most erosive acidic beverages for diet counseling.

Unfavorable changes due to chronic exposure of teeth to acidic beverages do not correlate with a single drink consumption but denote a cumulative effect of consumption of these dietary acids on a daily basis, i.e., deleterious effects seen in teeth exposed to acidic beverages for 15 minutes per day for 96 days through *in vitro* studies correspond to the detrimental changes that would be observed after 25 years in individual's life time who consumes the beverage with the same frequency and duration. 7 10 11 12 13

Commercially available fruit juices and carbonated drinks were considered for this study since they adequately represent the diet habits of the current young generation. To our knowledge, the effect of acidic beverages on both the enamel and dentin at macroscopic and microscopic levels has not been analyzed yet in the same study. The effect of dietary preservatives on enamel and dentin is also not established. The present study has addressed the above concerns making it unique.

The acidic challenge preceded by food consumption on enamel erosion and discoloration different drinks impose on tooth color are challenging aspects to overcome. 14 15

In the current study, the discoloration potentials of various acidic beverages were scored and analyzed by using a customized scoring criterion in comparison with normal control. It was found that Coca-Cola, Mountain Dew, and Red Bull had maximum discoloration capacity. The discoloration caused by these agents can be attributed to the coloring agent present in these drinks. The key ingredients of Coca-Cola include carbonated water, sugar (sucrose or high-fructose corn syrup (HFCS), caffeine, phosphoric acid, and class 4—caramel color 150d /E150d. The ingredient responsible for staining is the class 4—caramel color 150d/E150d which is a dark brown coloring agent. 16 Mountain Dew is composed of carbonated water, HFCS, concentrated orange juice, citric acid, natural flavors, sodium benzoate, caffeine, sodium citrate, erythorbic acid, gum arabic, ethylenediaminetetraacetic acid, brominated vegetable oil, and tartrazine. Tartrazine is a synthetic lemon yellow azo dye primarily used as a food colorant which is primarily responsible for the staining of teeth in Mountain Dew. 17 Red Bull energy drink contains caffeine, taurine, B vitamins (B3, B5, B6, and B12), and simple sugars (sucrose and glucose) in a buffer solution of carbonated water, baking soda, and magnesium carbonate. 18 Caramel produced by ammonia process along with riboflavin is a common food coloring agent used in Red Bull energy drink responsible for staining. 19

Erdemir et al 2016, in a recent study, demonstrated that energy drinks had a risk of causing discoloration due to their wide variety of ingredients. Varying amounts of caffeine, guarana extract, taurine, and ginseng are the main ingredients of energy drinks which are responsible for discoloration. 20 The mean loss of weight in the tooth structure can be attributed to the degree of the demineralization. In the present study, the maximum mean weight loss was observed with respect to vinegar followed by apple cider.

The explanation for the above result is that vinegar typically contains 5 to 20% acetic acid by volume. Usually, the acetic acid is produced by the fermentation of ethanol or sugars by acetic acid bacteria resulting in a lowered pH. 21 Apple cider is fermented juice from crushed apples and contains lactic acid, succinic acid, and acetic acid. Bacteria and yeast are added to the liquid to start the alcoholic fermentation process, which converts the sugars to alcohol. In a second fermentation step, the alcohol is converted into vinegar by acetic acid-forming bacteria (Acetobacter species). Acetic acid and malic acid combine to give vinegar its sour taste and a lowered pH. 22

Red Bull energy drink showed the maximum mean loss of weight among the carbonated drinks. The above results can be attributed to the pH of the acidic beverages used; vinegar had the lowest pH resulting in maximum mean loss followed by apple cider. Red Bull energy drink has 27 g of sugar per can. Taurine, or 2-aminoethanesulfonic acid, is an amino acid naturally made in the human body. Found in the lower intestine and a major component of bile, taurine is an antioxidant that helps to move minerals through the system and generate nerve impulses. Each can of Red Bull contains 1,000 mg of taurine. 23 These components can significantly lower the mineral component of hard tissues.

A study conducted by Mathew et al 2017 weighed enamel before and after immersion in different beverages. The mean weight reduction by orange juice was 21% followed by Red Bull (13%), Pepsi (11%), lemon juice (16%), apple juice (16%), coffee (3%), and green tea (3%). This demonstrated the erosive potential of these beverages. 24

Although the mean loss of weight denotes the demineralization, it is a quantitative value and does not describe the effects of these beverages individually on the enamel and dentin. To assess the erosivity of these drinks on the enamel and dentin, the current study used advanced microscopic methods.

The changes in the surface of the tooth structure also determine the strength and longevity of the tooth. These surface changes in the teeth specimens were observed using a stereomicroscope. Loss of gloss and roughness of the tooth structure cause more plaque accumulation and, hence, lead to demineralization, decay, and poor oral hygiene. 25 In the present study vinegar and apple cider showed maximum surface irregularities, loss of gloss and roughness, followed by lemon juice, Coca-Cola, and Mountain Dew. Grando et al 1996 reported the erosion caused *in vitro* by cola-type and guarana-type beverages (the latter is a soft drink sold in Brazil) and a canned lemon juice on the enamel of human deciduous teeth. Stereomicroscopic analysis showed loss of gloss and an alteration in the normal color of enamel, with irregular loss of dental tissue in variable degrees. The loss of gloss and roughness was directly proportional to the incubation time. 26 Both regular and sugar-free sodas also contain acids that have a demineralizing effect. With each sip of soda, a damaging reaction that lasts for approximately 20 minutes is initiated. 27

The present study used ground sections with thickness between 60 and 70 µm under 10x and 40x magnification. The parameters assessed with respect to the enamel and dentin were the integrity of striae of Retzius and dentinal tubules, respectively. Striae of Retzius was chosen since it represents the deposition of enamel and the enamel rods, which is the main component that determines the integrity of the enamel. Dentinal tubules were assessed since the integrity of the dentin is dependent on the configuration of these tubules. The present study is the first of its kind to analyze and correlate integrity of striae of Retzius and dentinal tubules with demineralization. Both parameters were given scores based on their appearance under the microscope. Maximum loss of integrity of striae of Retzius was observed in teeth exposed to apple cider and vinegar, followed by Coca-Cola. Lemon juice showed significant changes at 96 days. Although few studies have evaluated the effect of acidic beverages on human teeth, the current study for the first time has also evaluated the effect of dietary preservatives, the erosive effects of which are often neglected.

The daily consumption of apple cider vinegar seems to be trending among celebrities, especially women as the most popular oral supplement for weight reduction. 28 The health benefits of apple cider vinegar have been well documented in inducing weight loss in obese individuals. A recent study by Khezri et al 2018 has shown that apple cider vinegar intake along with a restricted diet helps in decreasing body weight plasma triglycerides and basal metabolic index. 29

Individuals consuming apple cider vinegar remain exposed to erosive damage to the enamel, greater damage is inflicted when consumed at night as the buffering action of saliva is absent. A case reported in 2012 strongly suggests that erosive tooth wear is easily induced by the daily consumption of apple cider vinegar. 30 The results of the current study serve as a word of caution for those frequently consuming apple cider vinegar.

The configuration of the striae of Retzius and dentinal tubules determines the orientation of the hydroxyapatite crystals of the enamel and dentin. 31 A change in this configuration results due to chronic exposure of the tooth structures to acidic beverages resulting in demineralization. As both enamel and dentin have inherent crystalline properties, their histological features are better visualized under a polarizing microscope than transmitted light microscopy since structures such as crystals and collagen fibers have the property to split polarized light into two, a phenomenon known as birefringence. 32 33 Hence, the degree of demineralization of the apatite crystals can be determined by the appearance of the enamel and dentin under the polarizing microscope.

Demineralization was observed as patchy and haphazard areas of birefringence with loss of stratification of colors. The degree and pattern of demineralization was found to be high once again for vinegar and apple cider closely followed by Red Bull and lemon juice. Coca-Cola and Mountain Dew had comparable values at 96 days. Literature review has shown that no study has employed polarizing microscopy to evaluate the demineralization of enamel and dentin by acidic beverages. White et al 2001 compared polarized microscopy findings of root caries and erosion of root structure caused by corrosive acids and stated that histological pattern of two zones of opposite birefringence is seen in all categories of erosive demineralization. 34 These zones of differing birefringence in dentin represent different degrees of mineralization of collagen.

## Conclusion

This present study is unique as it has utilized three different types of microscopes, i.e., bright field microscope, stereomicroscope, and polarizing microscope with additional assessment parameters of weight loss and discoloration making the analysis comprehensive.

We can conclude that vinegar and apple cider are the most detrimental dietary preservatives. Among the acidic beverage's lemon juice was the most harmful to the hard tissues. Coca-Cola and Mountain Dew were found to be potent demineralizing agents among the aerated drinks followed by red bull. Tomato ketchup showed significantly least weight change, surface irregularities, demineralization, dentinal tubules, and striae of Retzius changes. Thereby, we can infer that tomato ketchup shows the least detrimental effect on hard tissues.

With reference to the present study, oral health educators can reinforce important practices to frequent acidic beverage consumers such as decreasing the time that the beverage remains in the mouth and decreasing the frequency of consumption of these acidic beverages in a day. The use of regular fluoride application to convert the hydroxyapatite crystals to fluorapatite crystals will make the teeth resilient to demineralization. 33 The use of buffering agents and mouth rinses after consumption of acidic beverages can also be advocated that will help neutralize the acidic pH in the oral cavity.
